# Flotation Immunoassay: Masking the Signal from Free Reporters in Sandwich Immunoassays

**DOI:** 10.1038/srep24297

**Published:** 2016-04-14

**Authors:** Hui Chen, Anna E. V. Hagström, Jinsu Kim, Gavin Garvey, Andrew Paterson, Federico Ruiz-Ruiz, Balakrishnan Raja, Ulrich Strych, Marco Rito-Palomares, Katerina Kourentzi, Jacinta C. Conrad, Robert L. Atmar, Richard C. Willson

**Affiliations:** 1 University of Houston, Department of Biology and Biochemistry, Houston, TX 77204, USA; 2 University of Houston, Department of Chemical and Biomolecular Engineering, Houston, TX 77204, USA; 3 Tecnológico de Monterrey, Departamento de Biotecnología e Ingeniería de Alimentos, Monterrey, Nuevo León 64849, Mexico; 4 Baylor College of Medicine, Department of Pediatrics, Houston, TX 77030, USA; 5 Baylor College of Medicine, Department of Medicine, Houston, TX 77030, USA

## Abstract

In this work, we demonstrate that signal-masking reagents together with appropriate capture antibody carriers can eliminate the washing steps in sandwich immunoassays. A flotation immunoassay (FI) platform was developed with horseradish peroxidase chemiluminescence as the reporter system, the dye Brilliant Blue FCF as the signal-masking reagent, and buoyant silica micro-bubbles as the capture antibody carriers. Only reporters captured on micro-bubbles float above the dye and become visible in an analyte-dependent manner. These FIs are capable of detecting proteins down to attomole levels and as few as 10^6^ virus particles. This signal-masking strategy represents a novel approach to simple, sensitive and quantitative immunoassays in both laboratory and point-of-care settings.

Sandwich immunoassays have been widely used in biomedical diagnosis, food safety analysis, and environmental monitoring^1^. Two essential steps are involved: formation of an antibody-target-antibody:reporter sandwich, and removal of excess reporters. In the first step (Fig. 1A1), capture antibodies, analytes and reporters form sandwich structures. Excess capture antibodies and reporters are usually applied in this step to ensure every analyte participates in the sandwich structures. In the second step (Fig. 1A2), excess free reporters are removed by washing steps. However, removing the free reporters also affects the association/dissociation equilibrium between reporters and analytes^2^, thereby decreasing the fraction of analytes labelled with reporters and raising the limit of detection (LOD). This phenomenon is especially significant when low affinity detection antibodies are used^3^. The laborious and time-consuming washing process also is susceptible to errors, and produces potentially-infectious waste. The goal of this work is to eliminate the need to remove free reporters through traditional washing steps, and thereby build a more user- and environment-friendly assay platform.

The purpose of removing free reporters in traditional sandwich immunoassays is to eliminate the nonspecific signal originating from these free reporters. If free reporters were not to generate a signal, or if their signal could be blocked, washing would become obsolete. Recently, a large body of work has explored the first strategy by developing novel, conditionally-responsive reporters which do not generate any signal in their free state, but only produce a signal in the proximity of an antibody-target sandwich. Such assays include fluorescence polarization (FP) assays^4^, fluorescence resonance energy transfer (FRET) or time resolved FRET (TR-FRET) assays^5^, singlet oxygen-induced luminescence proximity assays^6^, electrochemiluminescence assays^7^, assays based on enzyme fragment complementation of *β*–galactosidase^8^, and acridan-based chemiluminescence^9^ assays. However, these assays usually require exotic reporters or proprietary/specialized instruments. Since most of these proximity assays require the donor-acceptor distance to be between 1–10 nm, they are usually not suitable for the detection of larger analytes such as viruses or bacteria.

An alternative strategy, the use of signal-masking reagents to block the signal from free reporters (Fig. 1A3), is explored for the first time in this work. We introduce familiar light-absorbing dyes as signal-masking reagents to block the light signal from free light-emitting reporters in sandwich immunoassays. As described by the Beer-Lambert law, the intensity of light decreases logarithmically along the light path^10^. Therefore, when appropriate dyes are present in solution, only the light-emitting reporters in the outermost layer, facing the detector, are detectable (Fig. 1B). In our approach, carriers modified with capture antibodies collect the light-emitting reporters involved in the immuno-sandwich structures and move them into the outermost layer, while leaving free reporters dispersed and undetectable in the bulk of the dyed solution. The amount of detectable reporters is proportional to the amount of analyte in the solution. In this way, traditional immunoassays can be upgraded to wash-free assays, without the need for novel, potentially exotic reporters or specialized instrumentation. Additionally, since this strategy does not rely on molecular-scale proximity, it can be applied to the detection of relatively large analytes such as cells and viruses.

## Results and Discussion

Horseradish peroxidase-chemiluminescence (HRP-CL), a popular and sensitive reporter system^11^,^12^,^13^,^14^, was chosen as the model light-emitting reporter system in this work. HRP molecules catalyze CL substrate oxidation and the concomitant light emission only in their immediate vicinity^15^. Each HRP molecule, therefore, can be regarded as an individual light emitter. For the specific substrate used in this work (FemtoGlow™), the major emission peak is around 460 nm (Fig. 2A). Therefore, a good signal-masking dye for HRP-CL should absorb strongly at this wavelength. Moreover, as most light-absorbing dyes are also fluorescent, and most light detectors are sensitive to light over a relatively wide spectral range (e.g. the plate reader used in this work is responsive to light from 380–600 nm), an ideal signal-masking dye should also avoid any fluorescence emission overlapping with the detection wavelength range of the detector. Based on these considerations, after comparing several widely used food coloring dyes we chose Brilliant Blue FCF (BB-FCF) as the proof-of-concept signal-masking reagent for the HRP-CL reporter system (Fig. 2B). Although other dyes, such as tartrazine, have better absorption properties around 460 nm, they also emit significant fluorescence in the detection range of the plate reader used; detection over a narrower range also could be used to resolve this issue.

We chose silica micro-bubbles as the immuno-carriers to collect labelled reporters at the top layer of the dyed solution. These are buoyant hollow glass microspheres with diameters ranging from 1 micron to several hundred microns. They offer high strength, low density, and very low cost^16^. Recently, due to their high surface to volume ratios (Fig. 3A,B), silica micro-bubbles have been introduced as immuno-carriers to the bioanalysis field^17^. Micro-bubbles can be easily delivered to the top layer of a solution by simple buoyancy, without any accessory devices to provide extra force fields. This property not only simplifies the assay process but also increases the robustness of the assay. In addition, the accumulation of the silica micro-bubbles at the top layer also blocks up to 40% of the unwanted luminescent emission from the lower bulk solution, adding to the effect of the dye (Fig. S1).

In this work, the silica micro-bubbles were silanized with triethoxysilyl butyraldehyde (TESBA), and the silane aldehyde groups were coupled to the primary amines of the proteins to be immobilized (antibody, NeutrAvidin, or bovine serum albumin (BSA)) by reductive amination. To confirm that proteins were successfully immobilized on the micro-bubbles by this method, both NeutrAvidin-modified and BSA-modified silica micro-bubbles were incubated with biotinylated fluorescence reporters (biotin-M13-Alexa555), washed and then observed by fluorescence microscopy. Only the NeutrAvidin-modified particles showed strong fluorescence (Fig. 3C–F), indicating that the NeutrAvidin had been successfully attached and remained functional on the particles.

**Figure 1:**
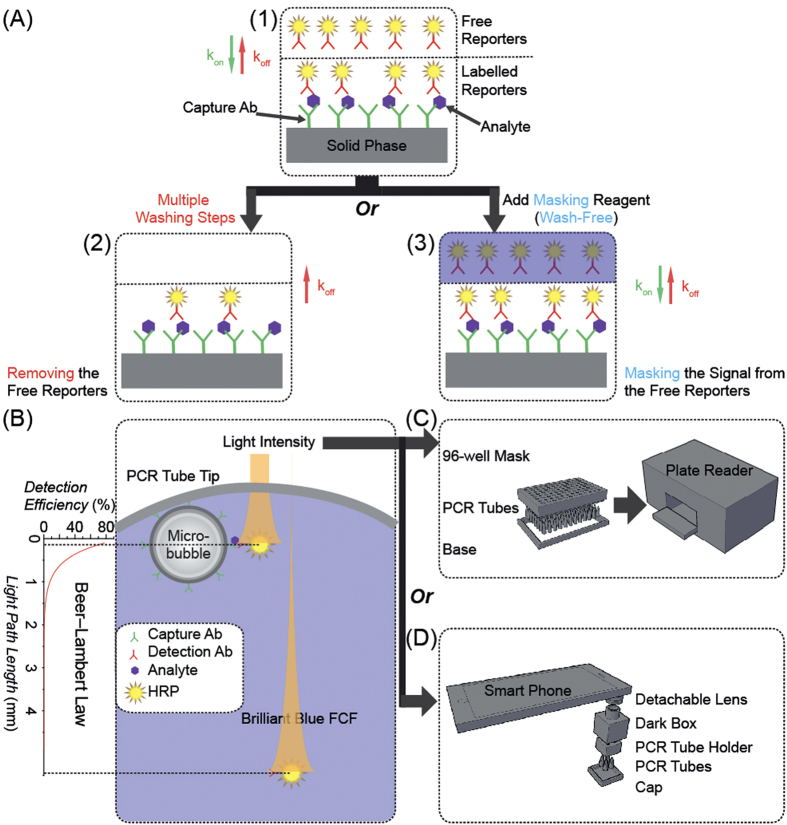
(**A**) Working principle of the signal-masking strategy for wash-free, flotation immunoassay (FI). 1) In a sandwich immunoassay, excess reporters are used to drive efficient labelling of the analytes. 2) In traditional sandwich immunoassays, free reporters are removed by multiple washings, also resulting in a decrease of the fraction of labelled analytes. 3) In the signal-masking based sandwich immunoassay, free reporters are not removed, instead, their signal is masked, without influencing the degree of labelling of the analytes. (**B**) Working principle of the flotation immunoassay (FI). Light-absorbing dyes are used to block the light from the free reporters dispersed in the bulk of the solution. Here, horseradish peroxidase (HRP) is used as the light-emitting reporter; Brilliant Blue FCF dye (BB-FCF) is used as the light-masking reagent; and silica micro-bubbles are used as the immuno-carriers to collect the labelled HRP molecules in the top detectable layer of the solution. (**C**) Design of the FI plate for plate reader read-out. (**D**) Design of the FI accessory for smart phone read-out.

**Figure 2:**
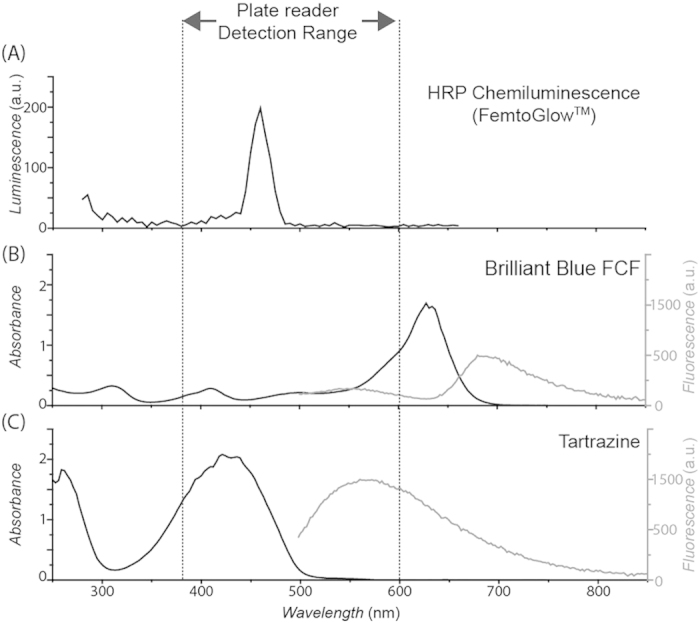
Selection of Brilliant Blue FCF dye (BB-FCF) as the signal-masking reagent. (**A**) Chemiluminescence emission spectrum of horseradish peroxidase (HRP) with FemtoGlow™ substrate; (**B**,**C**) Absorbance spectrum (black lines) and fluorescence emission spectrum (gray lines, excited at 460 nm) of (**B**) BB-FCF and (**C**) tartrazine. Vertical dashed lines indicate the luminescence detection range of the plate reader used in this work. The absorbance of BB-FCF is not as strong as those of some other dyes, but its fluorescence emission lies outside the range of sensitivity of the plate reader.

**Figure 3:**
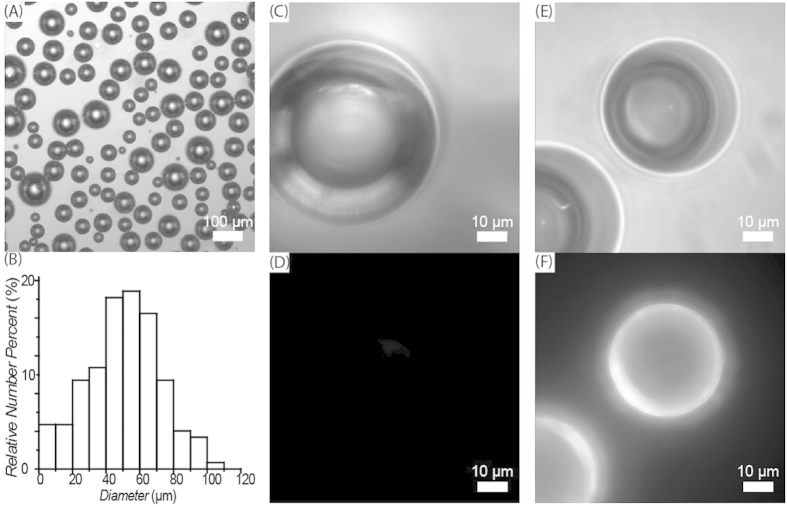
(**A**) Bright field image of micro-bubbles. (**B**) Size distribution of micro-bubbles, measured by microscopic imaging. (**C**) Bright field and (**D**) fluorescence images of BSA modified micro-bubbles labelled with biotinylated fluorescent reporters (biotin-M13-Alexa555). (**E**) Bright field and (**F**) fluorescence images of NeutrAvidin modified micro-bubbles labelled with biotinylated fluorescent reporters (biotin-M13-Alexa555).

With HRP-CL as the reporter system, BB-FCF as the signal masking reagent and silica micro-bubbles as the immuno-carriers, we designed a simple and wash-free flotation immunoassay (FI) (Fig. 1B), and demonstrated that the cumbersome washing steps in traditional sandwich immunoassays could be replaced by signal-masking reagents together with appropriate immuno-carriers. In this approach, silica micro-bubbles modified with capture antibodies and HRP tagged with detection antibodies were pre-mixed in standard polymerase chain reaction (PCR) optical tubes. The only end-user intervention is to add the sample of interest to these tubes, incubate the tubes, add the CL substrate pre-mixed with BB-FCF (10:1), and read out the signal either by luminometry (e.g., a plate reader) or imaging (e.g., a smart phone). During the entire assay process, no washes or aspirations are required, and no potentially-infectious wash waste is generated.

**Figure 4:**
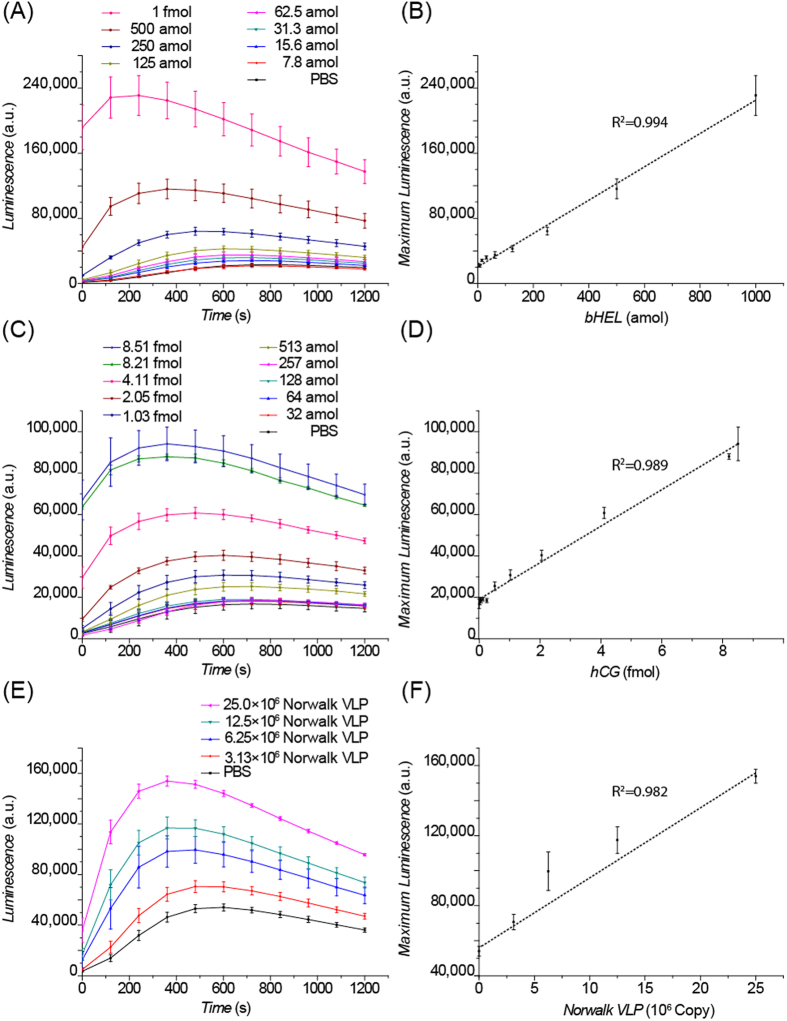
(**A**) Signal profiles of the Flotation Immunoassay (FI) for the detection of biotinylated lysozyme (bHEL). (**B**) Linear response of the maximum chemiluminescence (CL) signal to the amount of bHEL. (**C**) Signal profiles of the FI for human chorionic gonadotropin (hCG) detection. (**D**) Linear response of the maximum CL signal to the amount of hCG. (**E**) Signal profiles of the FI for Norwalk VLP detection. (**F**) Linear response of the maximum CL signal to the amount of Norwalk VLP. Mean ± standard deviation; n = 3.

To demonstrate the feasibility of FI, we used the detection of biotinylated hen egg lysozyme (bHEL). HEL is the canonical model system for physical immunochemistry and well-characterized, high affinity monoclonal antibodies are available, such as HyHEL-5^18^. In this assay, micro-bubbles were functionalized with HyHEL-5, and streptavidin-HRP was used as the reporter. To make the assay compatible with widely available ELISA plate readers, an assay plate with standard 96-well spacing was designed and 3D-printed to hold the PCR tubes optically-isolated and inverted in the reader (Fig. 1C, details of the holder are available in the supporting information). As shown in Fig. 4A,B, the luminescence signal increased linearly with the amount of bHEL, with an LOD of 15.6 amol (in 130 *μ*L solution, signal higher than the blank signal plus 3 standard deviations), demonstrating the feasibility of the washless flotation immunoassay.

The specificity of the assay for bHEL detection was then investigated with analogs of bHEL, including non-biotinylated HEL, and biotinylated BSA (bBSA). As shown in Fig. S2A, with the same amount of analytes tested (2 fmol), neither non-biotinylated HEL nor bBSA yielded significant signal increases, compared to the PBS control, while bHEL gave a more than tenfold signal increase. To further prove the signal was caused by the specific interactions between bHEL and the HyHEL-5 antibodies immobilized on the micro-bubbles, different amounts of free antibodies, as inhibitors, were introduced together with 2 fmol bHEL. As shown in Fig. S2B, the signal observed with 2 fmol bHEL was significantly inhibited when the amount of free HyHEL-5 reached 100 fmol. These experiments demonstrate that the observed FI signal increases were caused by specific molecular recognition of bHEL.The reproducibility of the assay is also good, as shown in Fig. S7, The intra- and interday coefficient of variation (n = 3), is 6.6%, and 8.6% at 20 amole bHEL and 4.0%, and 6.3% at 200 amole bHEL, respectively.

We next investigated the versatility of the assay by detecting human chorionic gonadotropin (hCG) and Norwalk virus-like particles (VLP). hCG has been widely used for pregnancy testing and is also a biomarker for germ cell cancers of the ovaries and testes^19^. In our hCG detection scheme, polyclonal antibodies recognizing the *α*-subunit of hCG were immobilized on the surface of micro-bubbles; monoclonal antibodies recognizing the *β*-subunit of hCG were tagged with HRP molecules by biotin-streptavidin linkage. As shown in Fig. 4C,D, the luminescence signal increased linearly with the amount of hCG with an LOD of 513 amol. Preliminary experiments for detection of hCG in human serum (diluted 5-fold in PBS buffer) showed a similar LOD of 313 amol (Fig. S8). Given the sample volume of 10 *μ*L the estimated target concentration is 31 pM (11 mIU/mL); the claimed lower LOD for various commercial clinical tests is 10 mIU/mL hCG in serum^20^. The Norwalk virus is the prototype strain of noroviruses, one of the most common causes of gastrointestinal disease^21^. Because of the difficulty of cultivating norovirus *in vitro*^22^, non-infectious VLPs which morphologically and antigenically resemble the authentic virus particles are usually used in the development of diagnostics for Norwalk virus^23^. In our Norwalk VLP detection scheme, monoclonal Norovirus G1 antibodies were immobilized on the surface of the micro-bubbles, and secondary monoclonal Norovirus G1 antibodies (recognizing a different epitope) were tagged to HRP molecules via biotin-streptavidin linkage. As shown in Fig. 4E,F, the luminescence signal increased linearly with the amount of Norwalk VLP with an LOD of 3.13 × 10^6^ copies (in 130 *μ*L solution). FI, with its reduced potential for production of infectious aerosols and tolerance of turbidity, may be particularly useful for detection of highly-infectious viruses such as Norwalk in body fluids.

While luminometer-based CL detection measures overall luminescence intensity, imaging devices such as CCD and CMOS cameras are capable of yielding CL images with high spatial resolution, which may be more suitable for multiplex assays such as point-of-care (POC) tests and high-throughput screening. We investigated the compatibility of the bHEL flotation immunoassay with imaging by a cooled CCD-based laboratory high definition imager. As shown in Fig. S4, the CL intensity of the samples increased linearly with the amount of bHEL with an LOD of 15.6 amol (the same as the LOD with a conventional plate reader). These results demonstrate that the FI is compatible with imaging-based detection for sensitive and quantitative bioanalysis.

**Figure 5:**
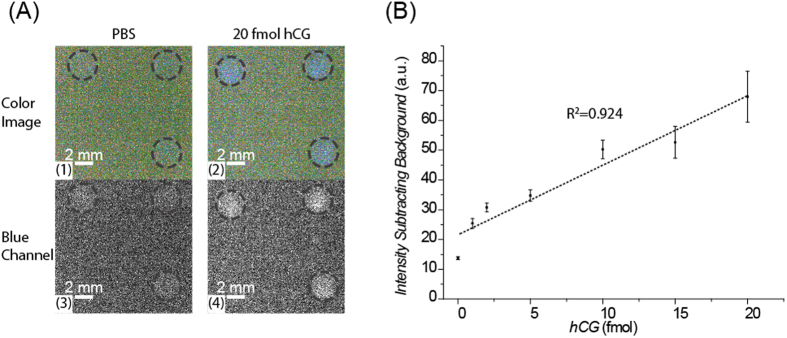
(**A**) Raw chemiluminescence images of the human chorionic gonadotropin flotation immunoassay (hCG FI), captured by a smart phone (iPhone 6 Plus) camera, using the SlowShutter app showing the color images of 1) PBS and 2) 20 fmol hCG and the isolated blue channel images of 3) PBS and 4) 20 fmol hCG. The PCR tubes holding the FI assay reagents are circled with dashed lines. (**B**) Quantitative analysis of the isolated blue channel images of the flotation immunoassay for hCG detection. Data represent the mean values ± the standard deviation obtained using three different PCR tubes.

Currently, smart phones enjoy great interest as POC analytical all-in-one devices to integrate analytical testing, data storage and information sharing for healthcare self-management^24^. To investigate the compatibility of our assay with smart phone imaging, we designed and 3D-printed an FI-compatible smart phone accessory (Fig. 1D, details are available in the supporting information). Since the HRP-CL signal is mainly around 460 nm, we isolated blue channel images from the smart phone for signal analysis. As shown in Fig. S5, there was an inherent unevenness of the brightness distribution in the background image of the smart phone. However, the brightness from a pre-determined position on the background image remained constant during consecutive image acquisitions. Therefore, the corresponding background brightness was subtracted from each image for CL signal analysis. Since the back-illuminated CMOS used in the smart phone (iPhone 6 Plus) is less sensitive than the cooled CCD in the laboratory HD imager^24^, we also increased the reporter level in the FI for smart phone imaging to achieve higher CL signals. As shown in Fig. 5, in a smart phone-based FI for hCG detection, the tubes with 20 fmol hCG showed significant light intensity while the PCR tubes with the PBS buffer showed only low background. Images of PCR tubes with other amount of hCG are available in the SI, Fig. S6. The light intensity increased linearly with the amount of hCG with an LOD of 1 fmol (in 130 *μ*L solution). These results demonstrate that FIs are also compatible with smart phone-based detection.

In conclusion, we have demonstrated for the first time that signal-masking reagents together with buoyant immuno-carriers can render the washing steps of currently existing sandwich immunoassays unnecessary, reducing effort and potentially-infectious waste. FI also uses common materials and equipment, and is compatible with larger targets such as bacteria and viruses. Finally we demonstrate that these assays can also be read by smart phones. Given their extreme simplicity of design and operation, the compatibility with low affinity recognition elements and multiple reading devices, the signal-masking-based FIs open up a new approach to simple, sensitive and quantitative biomedical assays in both laboratory and POC settings.

## Methods

### Optical Characterization of Reagents

The emission spectrum of HRP-catalyzed CL for the FI was recorded in a Tecan plate reader (Infinite^®^ M200 PRO, Tecan, Medorf, Switzerland; attenuation: none, integration time: 1 s, settle time: 0 ms). Twenty picograms of HRP in 5 *μ*L of PBS were mixed with 100 *μ*L FemtoGlow™ CL substrate. The luminescence spectrum was recorded from 280–660 nm; a major emission peak was detected around 460 nm.

The absorbance spectra of candidate dyes were recorded with a Nanodrop^®^ (ND-1000) spectrometer (Thermo Fisher Scientific, Inc. Rockford, IL, USA) with 2 *μ*L of 0.91% food coloring pigments (diluted in PBS). A lower concentration of Brilliant Blue FCF (0.91% here versus 9.1% in the FI) ensured absorbance values fit within the reader’s detection range. Absorbance was recorded from 280–660 nm with a 1 mm light path. The brilliant blue FCF shows an absorbance value of 0.12 at 460 nm. Using the Beer-Lambert law (*A* = *εcL*), we estimated the value of *ε*c for 9.1% brilliant blue FCF as 1.2 *mm*^−1^.

The fluorescence spectra of candidate dyes were recorded in a black 96-well plate with a transparent bottom in the Tecan plate reader. Fifty microliter of 0.91% dye (10 times diluted in PBS compared with the concentration used in FI) was used for the scans to fit into the reader’s detection range. Samples were excited at 460 nm and the emission was recorded from 500–850 nm.

The turbidity of the silica micro-bubble layer was investigated by comparing the luminescence readings of two sets of PCR tubes with or without a micro-bubble layer, using a Tecan plate reader (same luminescence settings as above). One PCR tube contained 1 *μ*L compacted micro-bubbles in PBS, 0.1 fmole HRP and 100 *μ*L CL substrate, while the other contained only 0.1 fmole HRP and 100 *μ*L CL substrate. As shown in Fig. S1, the 1 *μ*L micro-bubble layer blocked 40% of the chemiluminescent emission.

### Micro-bubble Preparation

Forty milligrams of micro-bubbles were washed with 5 N sulfuric acid for 1 h by slow inversion on a rotator (10 rpm), then washed 6 times with distilled water and 6 times with 95% ethanol. For silanization, micro-bubbles were resuspended in triethoxysilyl butyraldehyde (TESBA, 1:49 diluted in 95% ethanol) and reacted by slowly rotating on a rotator (10 rpm) for 30 min at 25 °C. The silanized micro-bubbles were then washed 5 times with pure ethanol and cured in pure ethanol for at least 1 hour at 25 °C followed by 5 additional washes with pure ethanol and 8 washes with filtered PBS. Then the micro-bubbles were added to 1 mL of PBS with 0.5 *μ*M protein (antibodies/NeutrAvidin/BSA) and 100 mM sodium cyanoborohydride (NaBH3CN). The modification reaction was kept at 4 °C with slow rotation (10 rpm) for 48 hours, after which the micro-bubbles were washed 8 times with 0.1% BSA in PBS. The protein-modified micro-bubbles were then blocked with 0.1% BSA in PBS for 1 h at 4 °C with slow rotation (10 rpm) and then washed 3 times with 0.1 mg/mL BSA. The modified bubbles were stored at 4 °C with 0.1 mg/mL BSA. It was observed that the activity of antibodies was maintained on the micro-bubbles for at least 2 months.

To check whether protein was successfully attached to the surface of micro-bubbles, NeutrAvidin-modified micro-bubbles and BSA-modified micro-bubbles (1 *μ*L, compacted) were both mixed with 100 *μ*L of 1 × 10^9^ copies/mL of biotinylated fluorescent reporters (Biotin-Alexa555-M13) in 0.1 mg/mL BSA for 1 h and washed with 0.1 mg/mL BSA in PBS 6 times. Fluorescence microscopic images of the micro-bubbles were then taken (Leica DMI 3000B).

### Flotation Immunoassay (FI)

In the FI, 1 *μ*L of compact immuno-microbubbles was pre-mixed with 20 *μ*L of 100 pM (600 pM for smart phone based FI) HRP reporters in an optical PCR tube. For use, 10 *μ*L of sample was added to the PCR tube and incubated on a rotator at 10 rpm (10 min for bHEL detection, 30 min for hCG detection and Norwalk VLP detection).

Based on the concept of using the signal-masking reagent to replace the multiple washing steps in sandwich immunoassays, the simple FIs are inherently suitable for POC tests. Although in the laboratory FIs, we used a rotator in the incubation process to achieve maximum sensitivity, users can also choose manual shaking as an alternative for POC applications. As shown in Fig. S3, only 3 intervals of shaking (of 15 inversions each) at the beginning, 10 min and 20 min time points of a 30 min incubation process was sufficient to retain 67% of the luminescence signal in an FI for hCG detection.

After the incubation, 100 *μ*L of FemtoGlow™ CL substrate containing brilliant blue FCF (volume ratio, 10:1) was added to the PCR tube. The PCR tube was gently shaken (15 inversions) to mix the reagents inside. Then the CL signal was ready to be read. For reading by plate reader, the PCR tubes were inserted into the FI plate, after which the CL profiles were recorded with 2 min intervals. The maximum signals were obtained within 15 min after the addition of the substrate. For the laboratory HD imager-based reading, PCR tubes were inserted into the FI plate, which was imaged by the Alpha Innotech FluorChem HD imager (ProteinSimple Co. San Jose, CA, USA; 8 min exposure time, medium resolution/high sensitivity). For smartphone-based reading, a background image of the empty FI accessory was first captured with an iPhone 6 Plus using the SlowShutter app (low light mode, high exposure boost, shutter speed:15 s, picture resolution 8 MP, 30 fps) as reference, then the PCR tubes were inserted into the FI accessory, and imaged with the same settings.

## Additional Information

**How to cite this article**: Chen, H. *et al*. Flotation Immunoassay: Masking the Signal from Free Reporters in Sandwich Immunoassays. *Sci. Rep*. **6**, 24297; doi: 10.1038/srep24297 (2016).

## Supplementary Material

Supplementary Information

Supplementary Dataset
