# Supplementary material for: Flotation Immunoassay: Masking the Signal from Free Reporters in Sandwich Immunoassays
Source: Sci Rep. 2016 Apr 14;6:24297. doi: 10.1038/srep24297 (PMC4830940; doi:10.1038/srep24297)
Supplement: Supplementary Information [file srep24297-s1.pdf]

Flotation Immunoassay: Masking the Signal from Free Reporters in Sandwich Immunoassays

Hui Chen, Anna E. V. Hagström, Jinsu Kim, Gavin Garvey, Andrew Paterson, Federico Ruiz-Ruiz, Balakrishnan Raja, Ulrich Strych, Marco Rito-Palomares, Katerina Kourentzi, Jacinta C. Conrad, Robert L. Atmar, and Richard C. Willson*

1. Reagents and Materials

Silica micro-bubbles (K1 glass bubbles, mean diameter ~60 μm) were purchased from 3M Company (St. Paul, MN, USA). Albumin from bovine serum (BSA), streptavidin–horseradish peroxidase (HRP), human Chorionic gonadotropin (hCG, using the conversion factor 9.28 IU/μg from the WHO 3rd International Standard) and hen egg lysozyme (HEL) were purchased from Sigma-Aldrich, Inc. (St. Louis, MO, USA). Gold Seal™ cover glasses, Alexa Fluor 555 Carboxylic Acid (Succinimidyl Ester), EZ-link® sulfo-NHS-LC-Biotin and Zeba™ spin desalting columns were purchased from Thermo Fisher Scientific, Inc. (Rockford, IL, USA). Triethoxysilyl butyraldehyde (TESBA) was purchased from Gelest, Inc. (Morrisville, PA, USA). Brilliant blue FCF and Tartrazine food colorings were purchased from Kroger, Co. (Kroger® Assorted Food Colors, Houston, TX, USA). Polymerase chain reaction (PCR) optical tubes (8× strips) were purchased from Agilent Technologies, Inc. (Santa Clara, CA, USA). Phosphate Buffered Saline (PBS) tablets, pH 7.4 were purchased from Clontech Laboratories, Inc. (Mountain View, CA, USA). Ethyl Alcohol (200 proof) was purchased from Pharmco-AAPER, Inc. (Farmers Branch, TX, USA). Fluorescence and chemiluminescence (CL) 96 well plates were purchased from Greiner Bio-one, Inc. (Orlando, FL, USA). Sulfuric acid (98%) was purchased from EMD Millipore, Inc. (Darmstadt, Germany). FemtoGlow™ Horseradish Peroxidase CL (HRP-CL) substrate was purchased from Michigan Diagnostics, LLC. (Royal Oak, MI, USA). Mouse anti-lysozyme monoclonal HyHEL-5 antibodies were custom produced from hybridoma cells and purified by Protein G affinity chromatography by Biovest/National Cell Culture Center (Minneapolis, MN, USA). Mouse monoclonal Norovirus G1 antibody (#10-1510) and mouse monoclonal Norovirus G1 antibody (#10-1511) were purchased from Fitzgerald Industries International, Inc. (North Acton, MA, USA). *Escherichia coli*

biotin ligase (*birA*) was purchased from Avidity, LLC. (Aurora, Colorado, USA). Monoclonal anti-β hCG antibody (ABBCG-0402), and Goat anti-α hCG polyclonal antibody (ABACG-0500) were purchased from Arista Biologicals, Inc. (Allentown, PA, USA). An iPhone 6 Plus was purchased from Apple, Inc. (Cupertino, CA, USA). Anonymized human serum samples were obtained from the Gulf Coast Regional Blood Center (Houston, TX, USA).

2. Size Distribution of Micro-bubbles

The size distribution of the micro-bubbles was investigated by bright field microscopy. 1 μL of compacted micro-bubbles in PBS was diluted with a 5 μL PBS droplet on a glass slide. Two cover slips (0.2 mm) were placed on opposite sides of the droplet to restrict its movement. Another cover slip was placed on top of the droplet and the cover slips were gently moved to disperse the micro-bubbles evenly, thereby forming a single layer in the flat droplet. Three samples were prepared and 15 images were taken from each sample. The number and sizes of micro-bubbles in each image were counted and measured with NIH ImageJ 1.43U (Dr. Wayne Rashand, National Institutes of Health, USA).[1]

3. Preparation of Biotin-M13-Alexa555 phage particles

The biotinylated fluorescent phage reporters (biotin-M13-Alexa555) were constructed as previously reported.[2] In brief, 100 μL of 10^{12} / mL M13 phage were mixed with 20 μL PEG/NaCl (20% w/v PEG 8000/2.5 M NaCl) and incubated at 4 °C for 1 hr. The M13 phage solution was then centrifuged at 11,000 rpm for 20 min. After the supernatant was discarded by pipetting, the M13 phage pellet was resuspended with 100 μL of 0.2 M sodium bicarbonate buffer, pH 8.3. The 5 μL of 10 mg / mL Alexa Fluor 555 Carboxylic Acid (Succinimidyl Ester) was added to M13 phage solution (mole ratio, dye: phage ~ 240,000), followed by overnight incubation at 4°C on a shaker.

In order to deactivate uncoupled fluor molecules, 10 µL of 1.5 M hydroxylamine in PBS, pH 8.5 was added to the solution for 1 h at room temperature. To remove uncoupled fluor molecules, phage was first PEG precipitated as described above and then passed through a Zeba™ desalting column (7 KDa MW) according to the manufacturer's protocol. Fluorescently-labelled M13 particles were stored in the dark at 4°C until used. The M13 phage used here displays an AviTag peptide (a 15-amino-acid peptide that is a substrate for biotin ligase) on the phage tail protein, p3. The AviTag peptide are partially biotinylated during expression in *E. coli*, and can be more completely biotinylated *in vitro* through treatment of purified phage particles with the enzyme biotin ligase as we have previously reported.[3]

4. Protein Biotinylation and Antibody-HRP Conjugation

The biotinylation of BSA, HEL and antibodies was carried out with the EZ-Link sulfo-NHS-LC Biotin kit (Pierce), following the manufacturer's protocol. Briefly, the protein was mixed with sulfo-NHS-LC-Biotin in PBS pH 7.4 (mole ratios, 1:20 for HEL and BSA, and 1:5 for antibodies), and reacted at room temperature for 30 min. The uncoupled sulfo-NHS-LC-Biotin was removed with Zeba™ desalting columns (7 KDa MW) using the manufacturer's protocol. After the biotinylation, the mole ratio of biotin to proteins was estimated using 4′-hydroxyazobenzene-2-carboxylic acid (HABA) assay. The ratio for bHEL was ~2.3, for bBSA ~4.5 and for Abs ~1.1.

The biotinylated detection Abs were then conjugated to HRP through avidin-biotin linkage, by mixing with streptavidin-HRP at a mole ratio of 1:1 in PBS and reacted overnight at 4 °C.

5. 3D Design and Printing

The FI plate and the FI accessory for iPhone 6 Plus were designed using AutoCAD 2012 (Autodesk, Inc. San Rafael, CA, USA) and 3D printed in monochrome plastic resin, using a Stratasys

Dimension 1200 3D printer (Eden Prairie, MN, USA), at the UH NSMIT Technology Center (University of Houston, Houston, TX, USA).

The FI plate was designed in 96-well format with standard microtiter spacings to hold the PCR tubes optically-isolated and upside down. As shown in Scheme 1C, the FI plate consists of two parts, the PCR tube holder with 96 wells to hold the PCR tubes containing the reagents of the FIs, and the base for the adaption to the sample inlet of the plate reader. Each well in the PCR holder consists of two parts with different shapes. The top part is of a cylindrical shape, while the bottom part is in the shape of a standard PCR tube. The two parts are connected with a circular window with 3 mm diameter. A PCR tube is inserted into the well from the bottom part and chucked by it, with only one millimeter of its tip protruding into the top part of the well. The three mm high walls of the top parts ensure the protruding tips of the PCR tubes are optically-isolated from each other.

The FI accessory was designed to attach to an iPhone 6 Plus in alignment with the main camera. We used the Mpow clip-on detachable 10×lens (Mpow Co. Santa Clara, CA, USA) to achieve optimum focus. As shown in Scheme 1D, the accessory consists of one dark box to eliminate the influence of the environmental light, one PCR tube holder to hold the PCR tubes containing the FI reagents, a set of spacers to adjust the focus and a cap to seal the dark box. The accessory is locked to the Mpow lens, which is further attached to the phone with its clip-on design.

Original files of the 3D drawings for the 3D printed FI plate and smart phone FI accessory are available at the following URL:

https://drive.google.com/folderview?id=0B7XQKZ2u-Yg0fmIxcE9wNHZlaDhqQmdvWlR4NUFOUjl
OWERHYjJET3MxMC1Uc3BibmNTZjQ&usp=sharing

6. Norwalk virus-like particles

Norwalk virus-like particles (VLPs) were expressed, assembled and purified as reported previously.[4] Briefly, the major capsid proteins (VP1 and VP2) were expressed from a baculovirus vector in Sf9 insect cells. The VLPs were purified using a cesium chloride gradient, and the structure was confirmed by transmission electron microscopy. The VLPs were stored in PBS at 4 °C.

7. FI for hCG detection in human serum

1 µL of compact immuno-microbubbles was pre-mixed with 20 µL of 100 pM HRP reporters in an optical PCR tube. 10 µL of sample (hCG in 20% human serum in PBS) was added to the PCR tube and incubated on a rotator 10 rpm (30 min). All other experimental details were as described in the main text.

SI FIGURES

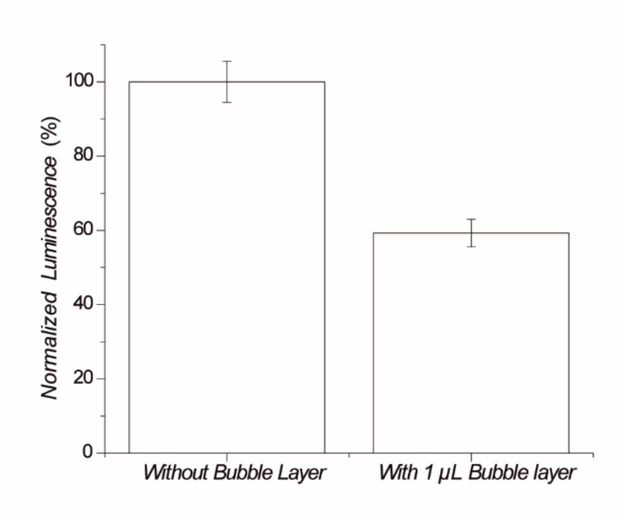

Figure S1. Optical blocking of the luminescent signal by 1 µL microbubble layer in PCR tubes. Data represent the mean values ± the standard deviation obtained using three different PCR tubes.

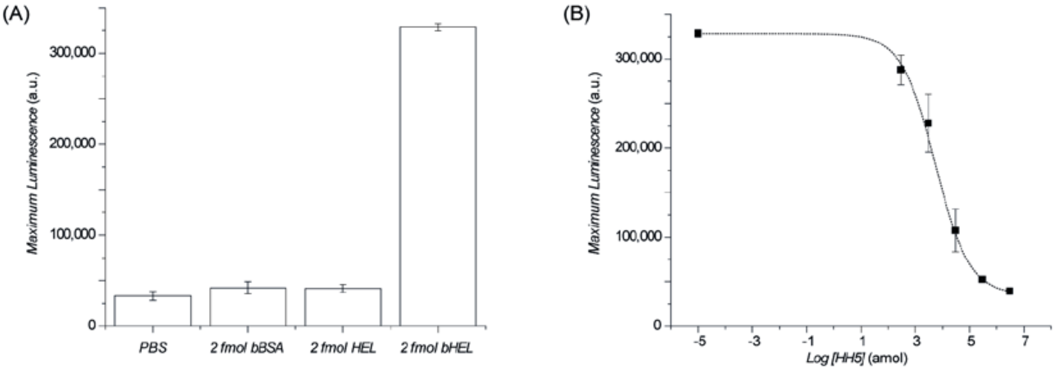

Figure S2. A) The selectivity of FI for biotinylated lysozyme (bHEL) detection. B) The inhibition effect of free HH5 anti-lysozyme antibody on the FI assay for bHEL detection. Data represent the mean values ± the standard deviation, n=3.

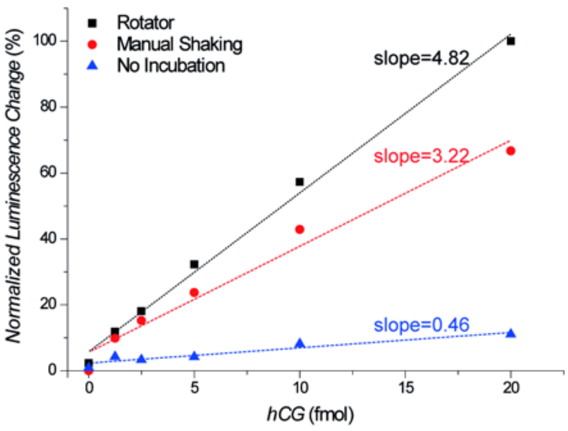

Figure S3. Comparison of the effect of different incubation methods on the FI for hCG detection. The black squares represent the FI with PCR tubes rotating (10 rpm) on a rotator for 30 min during the incubation process. The red dots represent the FI with the PCR tubes only manually shaken, 3 times at the beginning, and then again after 10 min and 20 min. The blue triangles represent the FI with no incubations.

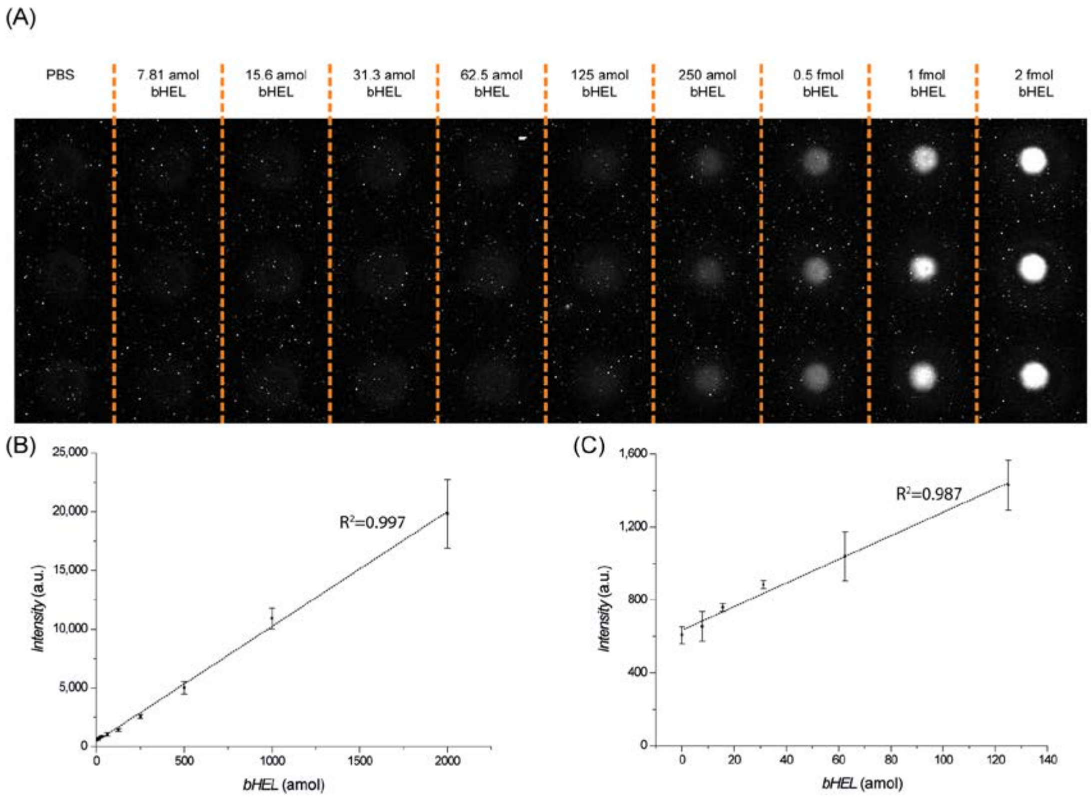

Figure S4. A) Raw CL images of the FI for bHEL detection, captured by a cooled CCD based HD imager, showing bHEL amounts from 0 to 2 fmole. B) and C) Quantitative analysis of the images of these images. Data represent the mean values ± the standard deviation, n=3.

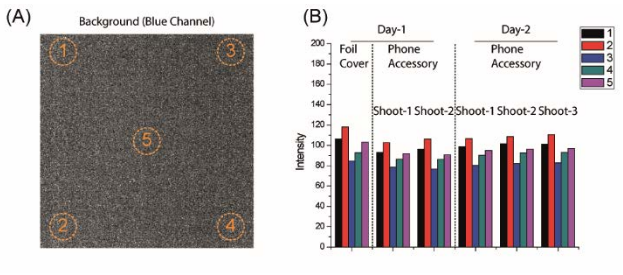

Figure S5. Study of the brightness distribution on the background image of the smart phone based FIs. A) The background image of the smart phone (iPhone 6 Plus) with its back camera attached to the unloaded FI accessory, captured by the SlowShutter app. The brightness of the 5 different positions on the background image, circled by the dashed lines, was investigated. B) Quantitative analysis of the brightness distribution of the smart phone background image. The five colors of the columns represent the five different positions on the background image. On Day 1, one background image was first taken with the back camera of the smart phone completely covered by foil, after which two other background images were consecutively shot with the camera attached to the unloaded FI accessory. On Day 2 three background images were consecutively shot with the camera attached to the unloaded FI accessory.

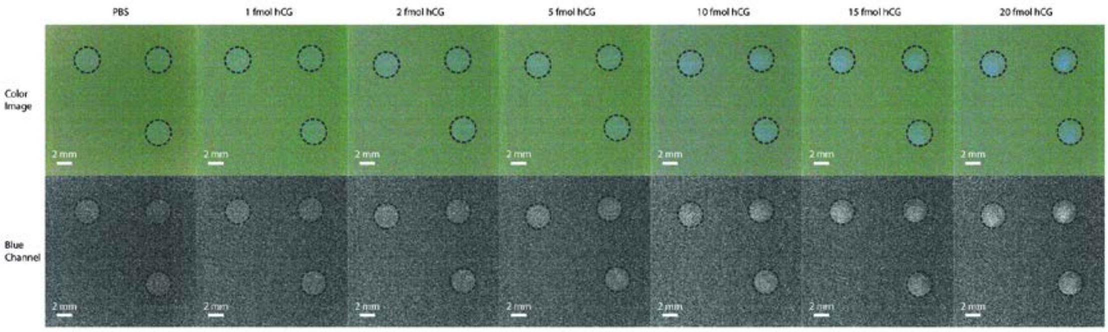

Figure S6. Raw CL images of the FI for hCG detection, captured by a smartphone camera, using the SlowShutter app showing the hCG amount from 0 to 20 fmol. The PCR tubes holding the FI reagents are circled with dashed lines.

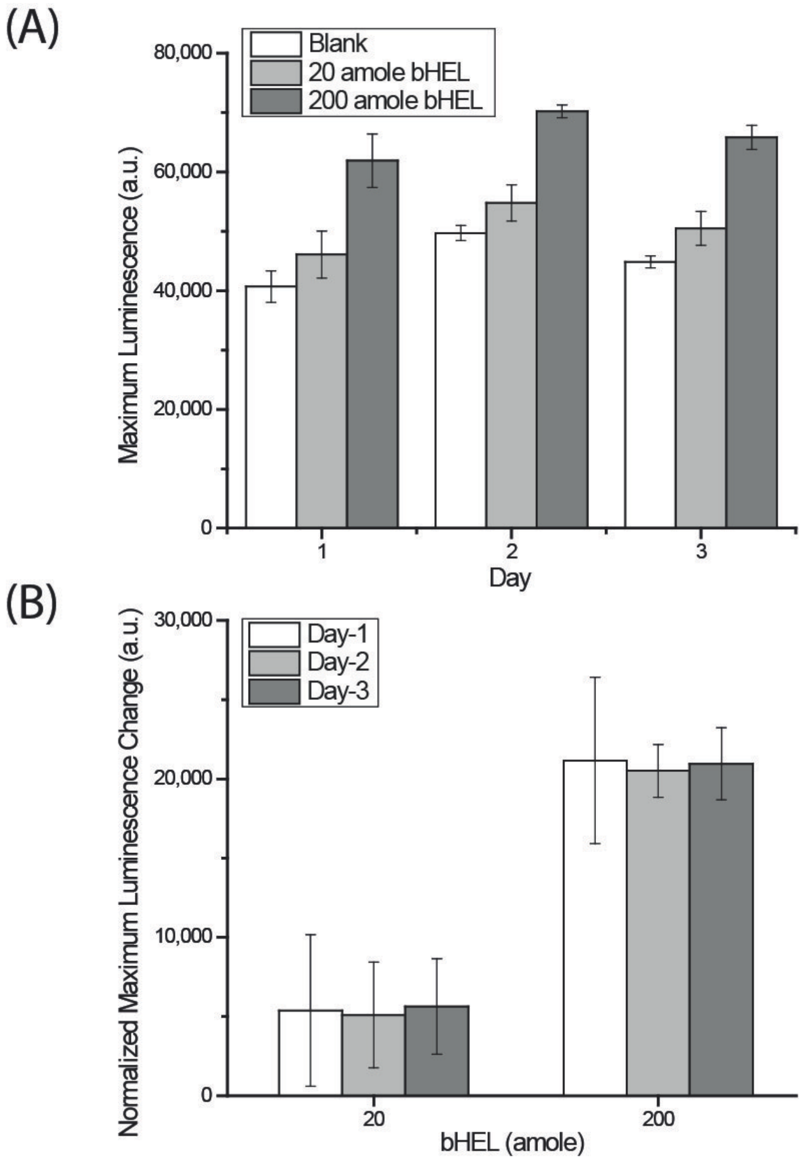

Figure S7. (A) Detection of bHEL with FI on three different days. The intra- and interday coefficient of variation (n = 3), is 6.6% and 8.6 % at 20 amole bHEL and 4.0% and 6.3% at 200 amole bHEL, respectively. (B) The signal at 20 amole bHEL and 200 amole bHEL in the three FIs are normalized to their corresponding blank signals. Mean ± standard deviation; n = 3.

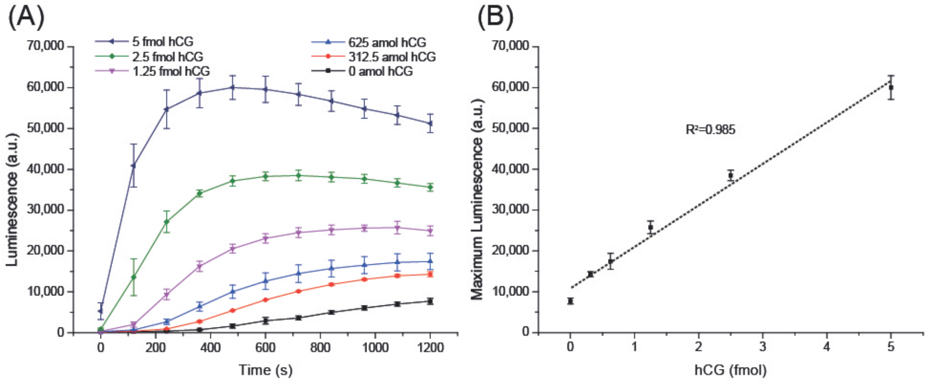

Figure S8. A) Signal profiles of the FI for human chorionic gonadotropin (hCG) detection in 20% human serum. D) Linear response of the maximum CL signal to the amount of hCG in 20% human serum. Mean ± standard deviation; n = 3.

References:

[1] W. Rasband, U. S. National Institutes of Health, Bethesda, MD, USA http://imagej.nih.gov/ij/, **1997-2014**.

[2] J. Kim, M. Adhikari, S. Dhamane, A. E. Hagstrom, K. Kourentzi, U. Strych, R. C. Willson, J. C. Conrad, *ACS Appl Mater Interfaces* **2015**, *7*, 2891-2898.

[3] M. Adhikari, U. Strych, J. Kim, H. Goux, S. Dhamane, M.-V. Poongavanam, A. E. V. Hagström, K. Kourentzi, J. C. Conrad, R. C. Willson, *Analytical chemistry* **2015**, *87*, 11660-11665.

[4] A. D. Hale, S. E. Crawford, M. Ciarlet, J. Green, C. Gallimore, D. W. Brown, X. Jiang, M. K. Estes, *Clinical and Diagnostic Laboratory Immunology* **1999**, *6*, 142-145.
